# Hearing Loss and Dementia: A Meta-Analysis of Prospective Cohort Studies

**DOI:** 10.3389/fnagi.2021.695117

**Published:** 2021-07-08

**Authors:** Zheng Liang, Ao Li, Yuanyuan Xu, Xiaoyun Qian, Xia Gao

**Affiliations:** ^1^Jiangsu Provincial Key Medical Discipline (Laboratory), Department of Otolaryngology Head and Neck Surgery, Affiliated Drum Tower Hospital of Nanjing University Medical School, Nanjing, China; ^2^Department of Neurology, The Fourth Affiliated Hospital of Nanjing Medical University, Nanjing, China

**Keywords:** hearing loss, dementia, Alzheimer's disease, prospective cohort studies, meta-analysis

## Abstract

**Background:** Consensus is lacking with regard to whether hearing loss is an independent risk factor for dementia. We therefore conducted a meta-analysis to clarify the relationship of hearing loss and dementia.

**Methods:** Prospective cohort studies investigating the association between hearing loss and the incidence of dementia in a community-derived population were included by searching electronic databases that included PubMed, Embase, and Cochrane's Library. A random-effects model was adopted to combine the results.

**Results:** Fourteen cohorts including 726,900 participants were analyzed. It was shown that hearing loss was independently associated with dementia [adjusted hazard ratio (HR): 1.59, 95% confidence interval (CI): 1.37 to 1.86, *p* < 0.001; *I*^2^ = 86%]. Sensitivity analysis sequentially excluding any of the individual studies included showed similar results. Subgroup analysis according to the diagnostic methods for hearing loss, validation strategy for dementia, follow-up duration, and adjustment of apolipoprotein E genotype also showed consistent results (*p*-values for subgroup differences all > 0.05). Meta-analysis with five studies showed that hearing loss was also connected to higher risk of Alzheimer's disease (adjusted HR: 2.24, 95% CI: 1.32 to 3.79, *p* = 0.003; *I*^2^ = 2%).

**Conclusions:** Hearing loss may increase the risk of dementia in the adult population. Whether effective treatment for hearing loss could reduce the incidence of dementia should be explored in the future.

## Background

Hearing loss is one of the most common sensory deficits in the elderly population (Andrusjak et al., 2020). A previous study in nationally representative data of the United States showed that more than half of the people over 60 years of age have a pure tone average (500, 1,000, 2,000, 4,000 Hz) > 25 dB and are classified as having hearing loss (Goman and Lin, 2016). With the accelerated progression of aging of the global population, it is estimated that more and more elderly people will suffer from hearing loss (Goman and Lin, 2018; Andrusjak et al., 2020). Studies have confirmed that hearing loss could adversely affect the physical and mental health of patients (Lohler et al., 2019). Moreover, hearing loss is a major cause of poor quality-of-life in the elderly population (Lohler et al., 2019). Besides, it has been suggested that hearing loss may be an important determinant of cognitive impairment (Jafari et al., 2019; Nixon et al., 2019). A previous meta-analysis including 40 studies published before 2016 showed that age-related hearing loss is significantly correlated with cognitive impairment (Loughrey et al., 2018). The authors also suggested that age-related hearing loss may be associated with higher risk of overall dementia, although such results were generally based on cross-sectional studies (Loughrey et al., 2018). In addition, results from experimental studies have also suggested some possible mechanisms linking hearing loss with dementia. For example, some potential mechanisms are the concurrent dysfunction of common pathways that cause hearing loss and dementia, as well as decreased stimulation of cognitive processing that results from hearing loss (Recanzone, 2018; Jafari et al., 2019; Uchida et al., 2019; Di Stadio et al., 2021). Accordingly, some prospective cohort studies have been conducted to reveal the relationship between hearing loss and the subsequent incidence of dementia (Gates et al., 1996, 2002, 2011; Lin et al., 2011; Gallacher et al., 2012; Gurgel et al., 2014; Deal et al., 2017; Golub et al., 2017; Amieva et al., 2018; Ford et al., 2018; Brenowitz et al., 2019; Osler et al., 2019; Vassilaki et al., 2019; Brewster et al., 2021). However, results of these studies were not always consistent. Therefore, a meta-analysis was conducted in this study to clarify the potential independent relationship between hearing loss and the subsequent incidence of dementia by combining the results of prospective cohort studies. In addition, the connection between hearing loss and Alzheimer's disease (AD) was also explored.

## Methods

The Meta-analysis of Observational Studies in Epidemiology (MOOSE) guideline (Stroup et al., 2000) and Cochrane's Handbook (Higgins and Green, 2011) were followed in this study.

### Literature Search

The electronic databases of PubMed, Embase, and Cochrane's Library databases were searched on January 23, 2021 with a strategy of combined terms (1) “hearing impairment” OR “hearing dysfunction” OR “hearing disorder” OR “hearing loss” OR “deaf” OR “deafness”; (2) “dementia” OR “cognitive decline” OR “cognitive impairment” OR “cognitive dysfunction” OR “Alzheimer” or “Alzheimer's”; and (3) “prospective” OR “prospectively” OR “longitudinal” OR “incident” OR “incidence” OR “risk” OR “followed” OR “follow-up” OR “cohort”. Only studies reported in English were considered. References of related articles or reviews were also analyzed.

### Study Identification

Studies that fulfilled these criteria were used: (1) prospective cohort studies published as full-length papers, (2) included general adult population, (3) evaluated the association between hearing loss and the incidence of dementia due to any cause or AD during follow-up, and (4) reported hazard ratios (HRs) for the above associations after adjusting for multiple confounding factors (at least for age and sex). Diagnostic criteria of hearing loss, dementia, and AD in the original articles were used. Reviews, preclinical studies, cross-sectional or retrospective studies, and irrelevant studies were not included.

### Data Extracting and Quality Evaluation

Two authors implemented database search, data extraction, and study quality assessment separately. If disagreements occurred, they were discussed with the corresponding author. These data were recorded: (1) author and study year; (2) participant characteristics, including number of participants included, mean age, and sex; (3) methods for the evaluation of hearing loss; (4) follow-up durations; (5) methods for validation of dementia or AD outcomes, and numbers of cases with outcomes reported in each study; and (6) potential confounding factors adjusted in the multivariate analyses. The Newcastle–Ottawa Scale (Wells et al., 2010) was used for study quality evaluation. This scale is rated from 1 to 9 stars and reflected the quality of the study by aspects of participants selection, comparability between groups, and outcome validation.

### Statistical Analyses

HRs and the corresponding 95% confidence intervals (CIs) were extracted for every included study. For studies that reported multiple HRs according to different models of multivariate regression analysis, the most adequately adjusted HR from each study was extracted and combined in this meta-analysis. Then, standard errors (SEs) of HRs were estimated from the 95% CIs or *p*-values. For normalization of their distribution, HRs were logarithmically transformed (Higgins and Green, 2011) and combined. Heterogeneity within the included cohort studies was tested via Cochrane's *Q*-test, as well as the estimation of I^2^ statistic (Higgins and Thompson, 2002). An *I*^2^ > 50% suggests significant level of heterogeneity. A random-effects model was chosen to combine the HRs by incorporating the potential heterogeneity within studies (Higgins and Green, 2011). Sensitivity analyses by sequentially excluding any of the individual studies included were conducted to clarify the influence of a certain study on the overall results (Patsopoulos et al., 2008). Predefined subgroup analyses according to the diagnostic methods for hearing loss, validation strategy for dementia, follow-up duration, and adjustment of the apolipoprotein E (APOE) genotype were also performed. Funnel plots were constructed and used for the assessment of publication bias (Egger et al., 1997). Visually asymmetrical funnel plots implied potential publication bias, which could be further validated by the Egger's regression asymmetry test. If high risk of publication bias was suggested, a “trim-and-fill” analysis was used for further evaluation, which estimates the influence of possible studies with negative findings on the meta-analysis outcome (Higgins and Green, 2011). The RevMan (Version 5.1; Cochrane Collaboration, Oxford, UK) and Stata software were involved for statistical analyses.

## Results

### Database Search

**Figure 1:**
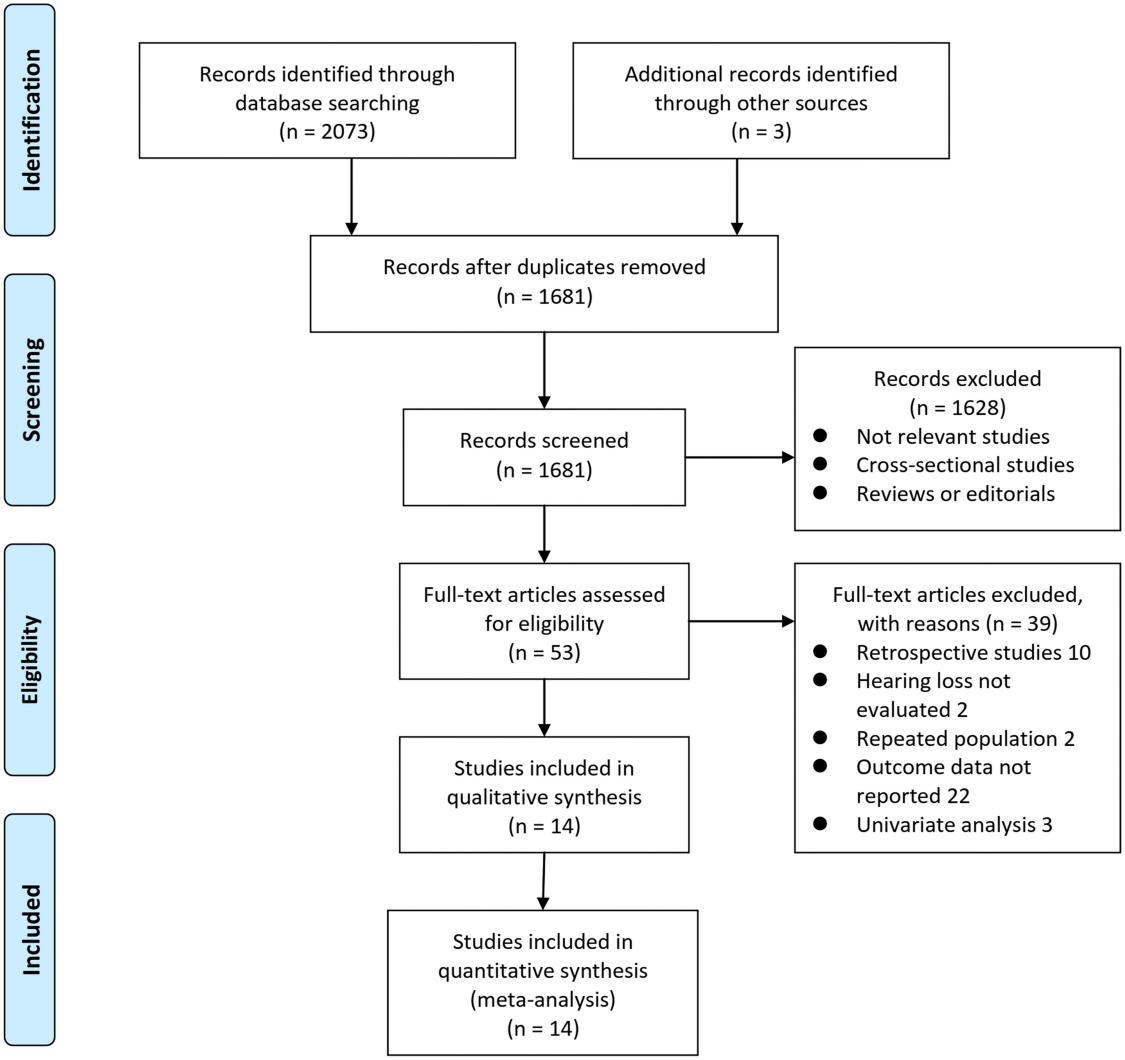
Scheme of study inclusion following PRISMA.

Details of the database search are shown in Figure 1. The first-step database search retrieved 1,681 articles after duplicated studies were excluded. Among them, 1,628 studies were further excluded because they were not related to the purpose of the meta-analysis based on titles and abstracts. Then, for the remaining 53 studies evaluated by full-text reading, 39 were not included for the reasons presented in Figure 1, which resulted in 14 prospective cohort studies finally analyzed in the meta-analysis (Gates et al., 1996, 2002, 2011; Lin et al., 2011; Gallacher et al., 2012; Gurgel et al., 2014; Deal et al., 2017; Golub et al., 2017; Amieva et al., 2018; Ford et al., 2018; Brenowitz et al., 2019; Osler et al., 2019; Vassilaki et al., 2019; Brewster et al., 2021).

### Study Characteristics

Characteristics of each study of the meta-analysis are shown in Table 1. Overall, 14 cohort studies including 726,900 participants were considered eligible for the meta-analysis (Gates et al., 1996, 2002, 2011; Lin et al., 2011; Gallacher et al., 2012; Gurgel et al., 2014; Deal et al., 2017; Golub et al., 2017; Amieva et al., 2018; Ford et al., 2018; Brenowitz et al., 2019; Osler et al., 2019; Vassilaki et al., 2019; Brewster et al., 2021), which were performed in the United States (Gates et al., 1996, 2002, 2011; Lin et al., 2011; Gurgel et al., 2014; Deal et al., 2017; Golub et al., 2017; Brenowitz et al., 2019; Vassilaki et al., 2019; Brewster et al., 2021), the United Kingdom (Gallacher et al., 2012), France (Amieva et al., 2018), Australia (Ford et al., 2018), and Denmark (Osler et al., 2019). All of the studies included a community-derived general population, and 11 of them included only an elderly population (Gates et al., 1996, 2002, 2011; Gurgel et al., 2014; Deal et al., 2017; Golub et al., 2017; Amieva et al., 2018; Ford et al., 2018; Brenowitz et al., 2019; Vassilaki et al., 2019; Brewster et al., 2021). Hearing loss was diagnosed via an audiometry test in 10 studies (Gates et al., 1996, 2002, 2011; Lin et al., 2011; Gallacher et al., 2012; Gurgel et al., 2014; Deal et al., 2017; Brenowitz et al., 2019; Osler et al., 2019; Brewster et al., 2021), but was also evidenced by self-reports (Golub et al., 2017; Amieva et al., 2018; Vassilaki et al., 2019) or International Classification of Diseases (ICD) codes (Ford et al., 2018). A total of 70,129 participants had hearing loss at baseline. With a mean follow-up duration between 4 and 44 years, 19,044 cases of dementia occurred. Diagnosis of dementia was based on clinical evaluation, which included medication use, hospital records, and neurocognitive test scores in 12 studies (Gates et al., 1996, 2002, 2011; Lin et al., 2011; Gallacher et al., 2012; Gurgel et al., 2014; Deal et al., 2017; Golub et al., 2017; Amieva et al., 2018; Brenowitz et al., 2019; Vassilaki et al., 2019; Brewster et al., 2021), and by ICD codes in the other two studies (Ford et al., 2018; Osler et al., 2019). Age, sex, education, comorbidities, and other potential confounding factors were adjusted to a varying degree when the associations between hearing loss and dementia were reported. The quality of these studies was good, evidenced by seven to nine points of NOS scores (Table 2).

**Table 1:** Characteristics of the included prospective cohort studies.

**References**	**Country**	**Participant characteristics**	**Sample size**	**Mean age**	**Male**	**Patients with hearing loss**	**Hearing loss diagnosis**	**Follow-up period**	**Outcome validation**	**Outcomes reported**	**Variables adjusted**	**NOS**
				**years**	**%**			**years**				
Gates et al. (1996)	USA	Community derived elderly population	816	72	45.3	191	SSI-ICM	8	Clinical evaluation	All-cause dementia (41)	Age and sex	7
Gates et al. (2002)	USA	Community derived elderly population	740	72	NR	177	SSI-ICM	9.7	NINCDS-ADRDA for AD	AD (40)	Age, sex, APOE4 allele, education, and PTA-WE	8
Gates et al. (2011)	USA	Community derived elderly population	313	79.6	37.2	58	SSI-ICM	4	NINCDS-ADRDA for AD	AD (21)	Age and educational level	7
Lin et al. (2011)	USA	Community derived population	639	63.1	56.3	184	Audiometry test	11.9	DSM-III-R for dementia, and NINCDS-ADRDA for AD	All-cause dementia (58), AD (37)	Age, sex, race, education, diabetes, smoking, and hypertension	9
Gallacher et al. (2012)	UK	Community derived male population	1,057	56.1	100	457	Audiometry test based on pure-tone unaided audiometric threshold	12.5	DSM-IV for dementia, and NINCDS-ADRDA for AD	All-cause dementia (79), AD (41)	Age, social class, anxiety, and premorbid intelligence	9
Gurgel et al. (2014)	USA	Community derived elderly population	4,463	75.8	56.1	836	Audiometry test as part of 3MS-R	12	DSM-III-R for dementia	All-cause dementia (575)	Age, sex, presence of ApoE4 allele, education, and cardiovascular risk factor	9
Golub et al. (2017)	USA	Community derived elderly population	1,881	75.8	30	204	Self-reported	7.4	DSM-III-R for dementia, and NINCDS-ADRDA for AD	All-cause dementia (377), AD (256)	Age, sex, ethnicity, race, years of education, diabetes mellitus, hypertension, heart disease, smoking, APOE4 genotype, and stroke	9
Deal et al. (2017)	USA	Community derived elderly population	1,889	75.5	47	1,103	Audiometry test	9	Dementia defined using a prespecified algorithm incorporating medication use, hospital records, and neurocognitive test scores	All-cause dementia (229)	Age, sex, race, education, study site, smoking status, hypertension, diabetes, and stroke	9
Amieva et al. (2018)	France	Community derived elderly population	3,588	75.3	42.2	1,289	Self-reported	25	Interview and neuropsychological evaluation, judged by a neurologist or geriatrician	All-cause dementia (576)	Age, sex, education, and comorbidities	8
Ford et al. (2018)	Australia	Community derived elderly male population	37,898	72.5	100	1,420	ICD codes	11.1	ICD codes	All-cause dementia (6,948)	Age, history of cardiovascular diseases, chronic respiratory diseases, diseases of the digestive system, kidneys, and cancer	7
Vassilaki et al. (2019)	USA	Community derived elderly population	4,812	73.7	51.5	981	Self-reported	5.4	Physician and neuropsychologist determined diagnosis	All-cause dementia (273)	Age, sex, and education	8
Osler et al. (2019)	Denmark	Community derived male population	658,465	NR	100	59,834	Audiometry test at interview	44.4	ICD codes	All-cause dementia (9,114)	Age, education, depression, diabetes, hypertension and cerebrovascular disease	8
Brenowitz et al. (2019)	USA	Community derived elderly population	1,810	77.1	51.2	1,344	Audiometry test at interview	10	Dementia defined using a prespecified algorithm incorporating medication use, hospital records, and neurocognitive test scores	All-cause dementia (336)	Age, race, sex, education, hypertension, diabetes, cardiovascular disease, cerebrovascular disease, smoking status, alcohol use, and physical activity	8
Brewster et al. (2021)	USA	Community derived elderly population	8,529	73.9	36.6	2,051	Audiometry test at interview	5.6	Dementia defined using a pre-specified algorithm incorporating medication use, hospital records, and neurocognitive test scores	All-cause dementia (498)	Age, sex, ethnicity, education, and APOE status	9

*NOS, the Newcastle–Ottawa scale; SSI-ICM, synthetic sentence identification-ipsilateral competing message; NINCDS-ADRDA, national institute of neurological and communicative disorders and stroke and the Alzheimer's disease and related disorders association; PTA-WE, speech frequency pure tone average of worse ear; DSM-III-R, diagnostic and statistical manual of mental disorders, 3rd edition revised for diagnosis of dementia; 3MS-R, modified mini-mental state exam-revised; AD, Alzheimer's disease; ICD, international classification of diseases; APOE, apolipoprotein E gene*.

**Table 2:** Details of quality evaluation via the Newcastle–Ottawa Scale.

**References**	**Representativeness of the exposed cohort**	**Selection of the non-exposed cohort**	**Ascertainment of exposure**	**Outcome not present at baseline**	**Control for age and sex**	**Control for other confounding factors**	**Assessment of outcome**	**Enough long follow-up duration**	**Adequacy of follow-up of cohorts**	**Total**
Gates et al. (1996)	1	1	1	1	1	0	1	1	0	7
Gates et al. (2002)	1	1	1	1	1	1	1	1	0	8
Gates et al. (2011)	1	1	1	1	1	1	1	0	0	7
Lin et al. (2011)	1	1	1	1	1	1	1	1	1	9
Gallacher et al. (2012)	1	1	1	1	1	1	1	1	1	9
Gurgel et al. (2014)	1	1	1	1	1	1	1	1	1	9
Golub et al. (2017)	1	1	1	1	1	1	1	1	1	9
Deal et al. (2017)	1	1	1	1	1	1	1	1	1	9
Amieva et al. (2018)	1	1	0	1	1	1	1	1	1	8
Ford et al. (2018)	1	1	0	1	1	1	0	1	1	7
Vassilaki et al. (2019)	1	1	0	1	1	1	1	1	1	8
Osler et al. (2019)	1	1	1	1	1	1	0	1	1	8
Brenowitz et al. (2019)	1	1	1	1	1	1	0	1	1	8
Brewster et al. (2021)	1	1	1	1	1	1	1	1	1	9

### Association Between Hearing Loss and Incident Dementia

**Figure 2:**
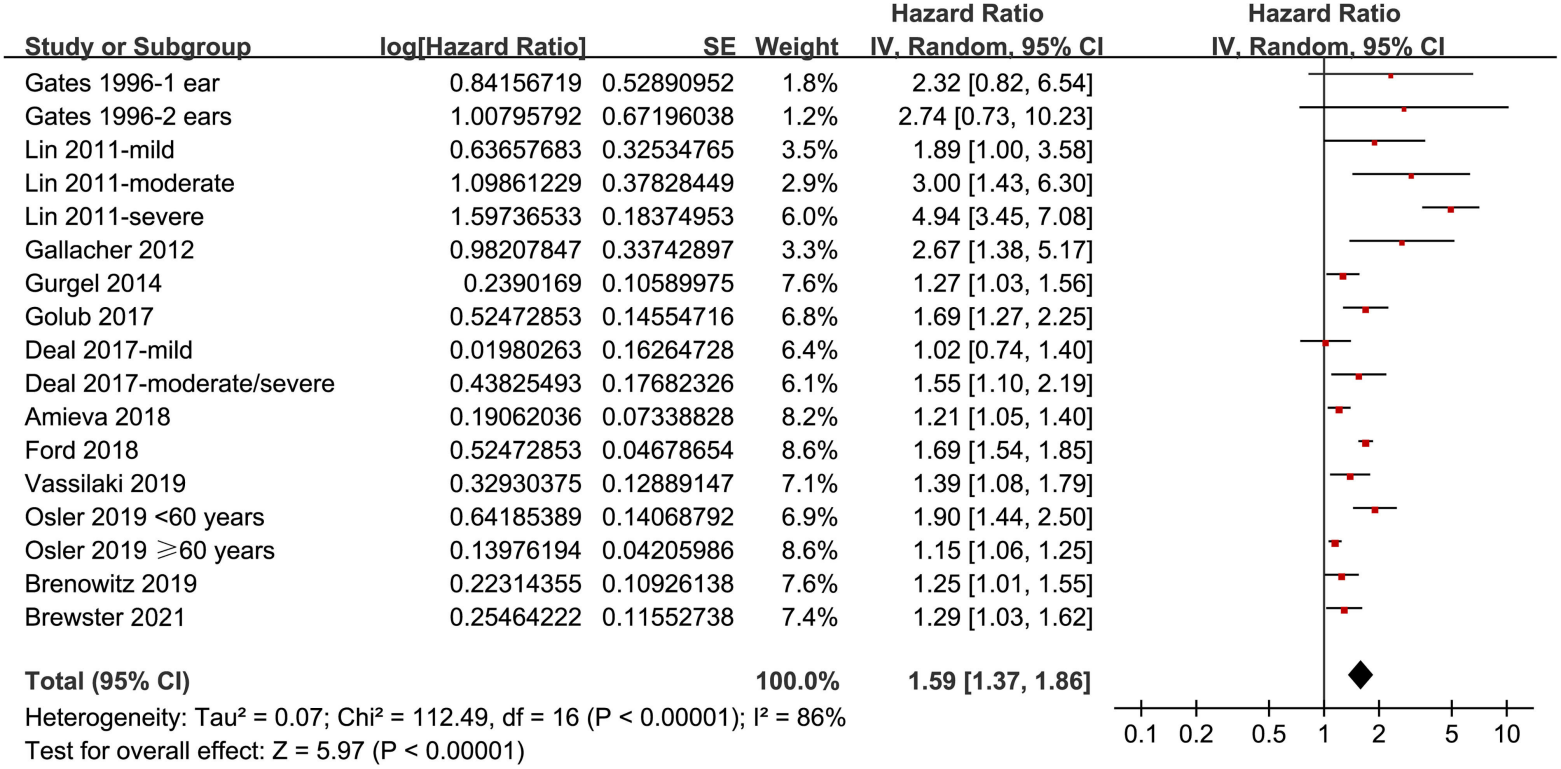
Forest plots for the meta-analysis concerning the association between hearing loss and the subsequent incidence of all-cause dementia.

**Figure 3:**
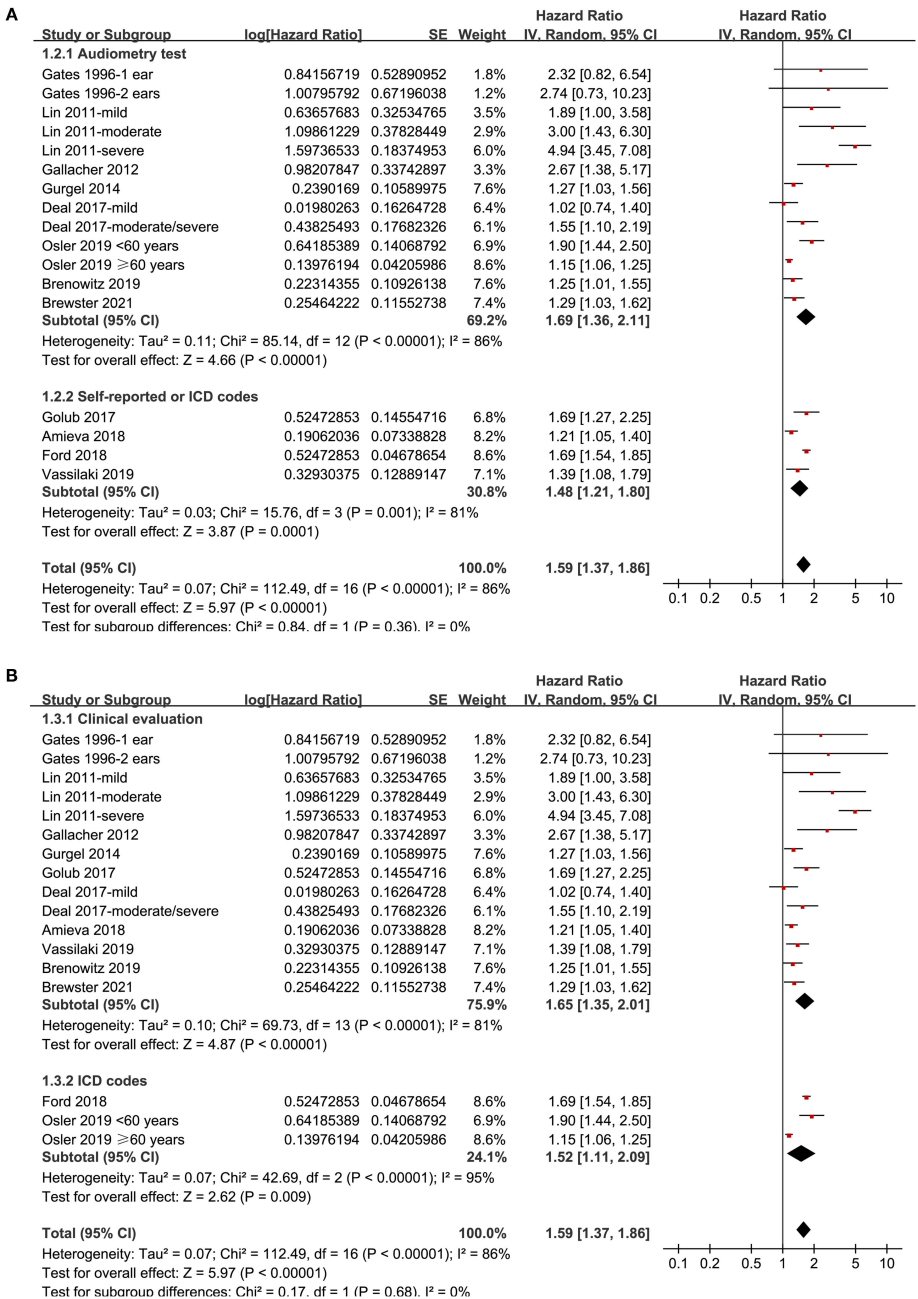
Subgroup analyses for the outcome of all-cause dementia. **(A)** Subgroup analysis according to the diagnostic methods for hearing loss, and **(B)** subgroup analysis according to the validation strategy for dementia.

**Figure 4:**
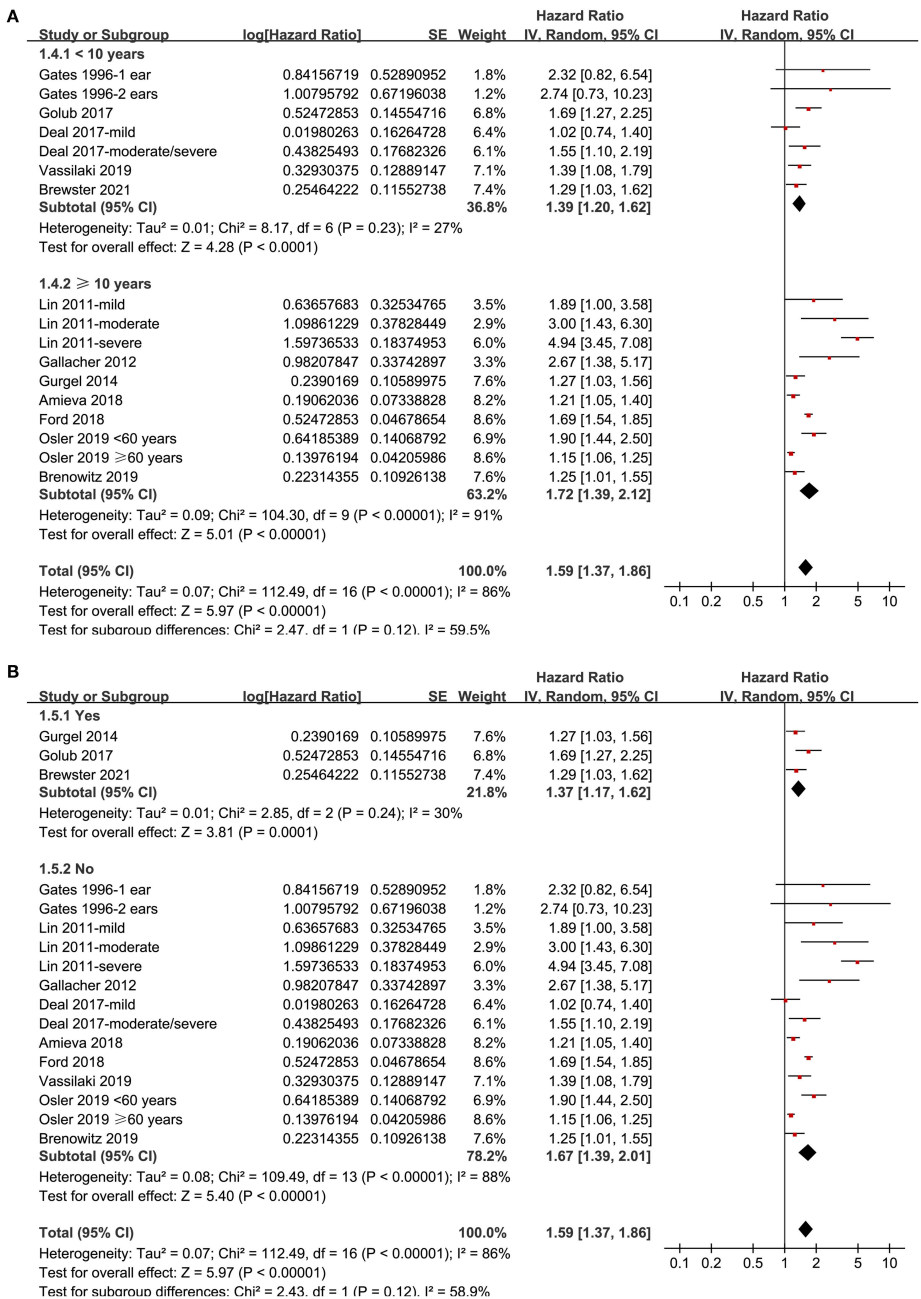
Subgroup analyses for the outcome of all-cause dementia. **(A)** Subgroup analysis according to the mean follow-up duration, and **(B)** subgroup analysis according to whether the status of the apolipoprotein E (APOE) genotype was adjusted.

**Figure 5:**
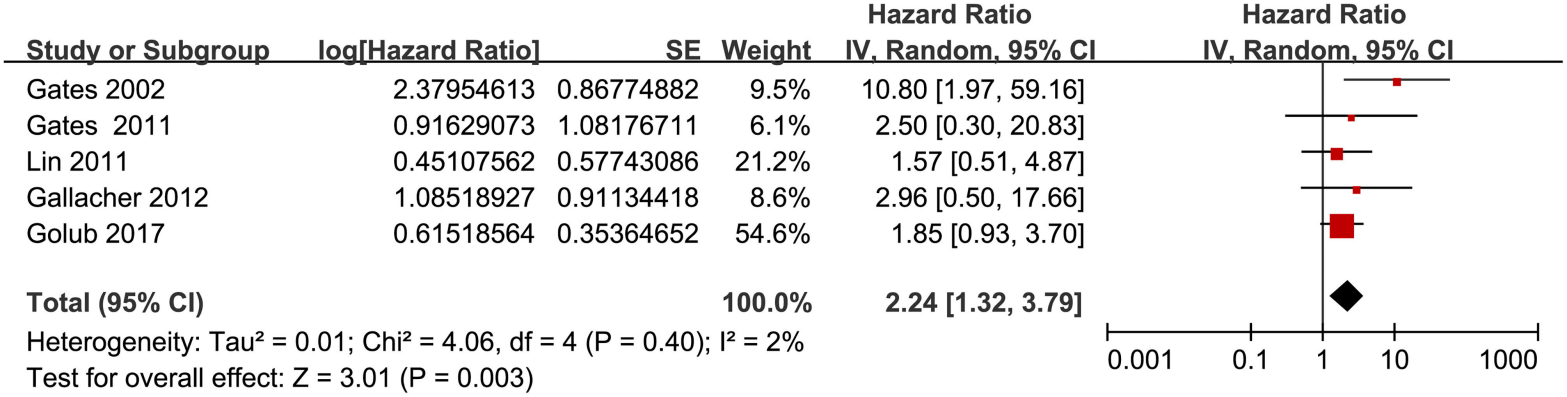
Forest plots for the meta-analysis concerning the association between hearing loss and the subsequent incidence of Alzheimer's disease (AD).

Twelve prospective cohort studies (Gates et al., 1996; Lin et al., 2011; Gallacher et al., 2012; Gurgel et al., 2014; Deal et al., 2017; Golub et al., 2017; Amieva et al., 2018; Ford et al., 2018; Brenowitz et al., 2019; Osler et al., 2019; Vassilaki et al., 2019; Brewster et al., 2021) were analyzed in the meta-analysis of hearing loss and risk of all-cause dementia. Since one study provided separate data according to the number of ears affected (Gates et al., 1996), two studies according to the severity of hearing loss (Lin et al., 2011; Deal et al., 2017), and the others according to the age of the participants (Osler et al., 2019), these datasets were independently examined. As a result, 17 datasets were available for the outcome of all-cause dementia (Figure 2). Pooled results showed that hearing loss elevated the risk of subsequent dementia [adjusted HR: 1.59, 95% confidence interval (CI): 1.37 to 1.86, *p* < 0.001; *I*^2^ = 86%; Figure 2]. Sensitivity analysis also showed consistent results (data not shown). Subgroup analysis by the diagnostic methods for hearing loss, validation strategy for dementia, follow-up duration, and adjustment of APOE genotype also showed consistent results (Figures 3, 4, *p*-values for subgroup differences all >0.05). Pooled analyses with five studies (Gates et al., 2002, 2011; Lin et al., 2011; Gallacher et al., 2012; Golub et al., 2017) showed that loss was independently associated with a higher incidence of AD (adjusted HR: 2.24, 95% CI: 1.32 to 3.79, *P* = 0.003; *I*^2^ = 2%; Figure 5).

### Publication Bias

**Figure 6:**
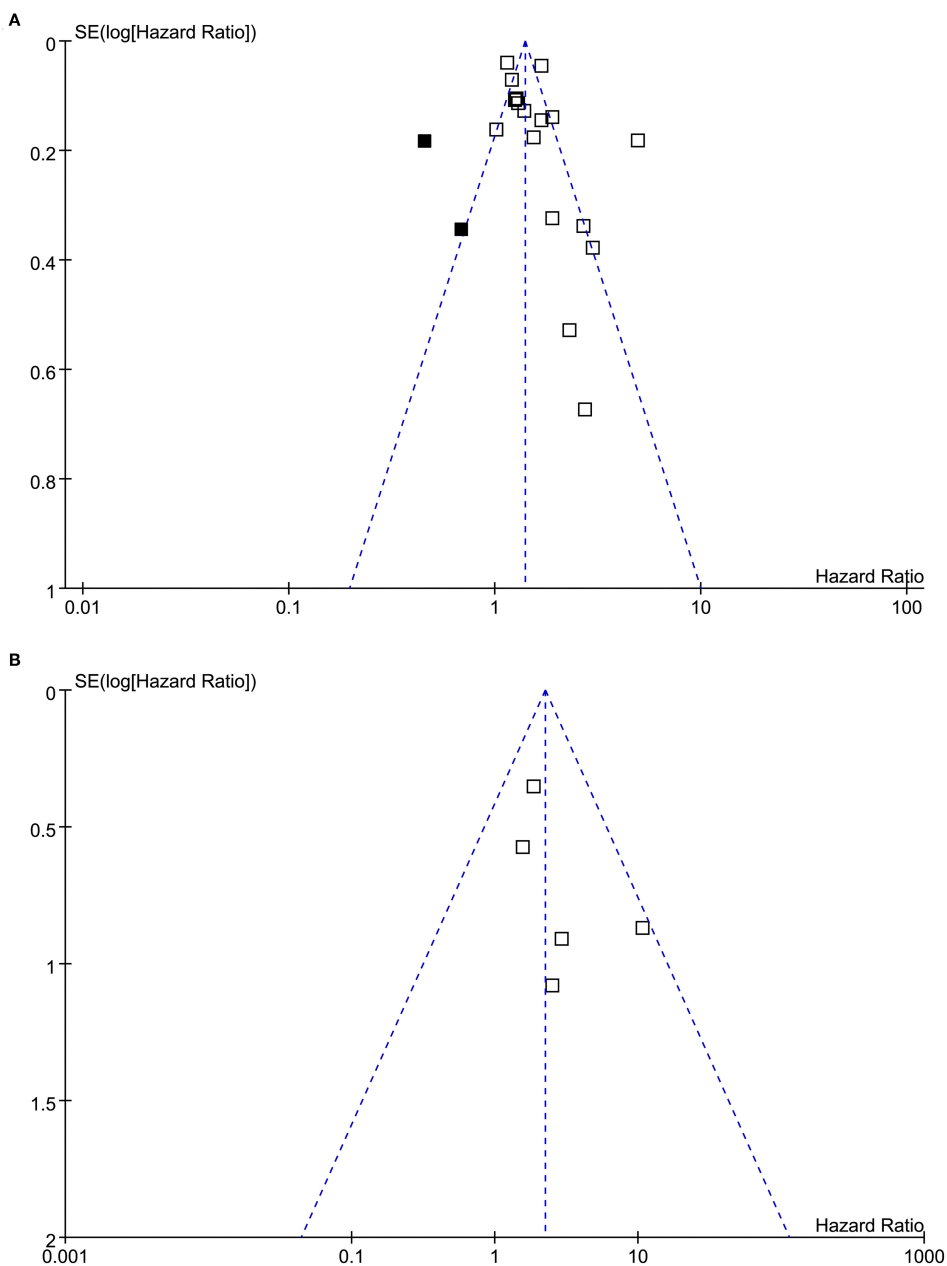
Funnel plots for the meta-analyses. **(A)** Funnel plots with trim-and-fill analysis for the meta-analysis concerning the association between hearing loss and the incidence of all-cause dementia (black square indicates hypothesized studies to achieve the symmetry of the funnel plots). **(B)** Funnel plots for the meta-analysis concerning the association between hearing loss and the incidence of AD.

Funnel plots representing the meta-analysis of hearing loss and all-cause dementia are shown in Figure 6A. The plots were asymmetrical based on visual inspection, raising the possible publication bias. Egger's regression test also demonstrated potential risk of publication bias. We therefore performed a trim-and-fill analysis. As shown in Figure 6A, incorporating the two hypothesized studies achieved symmetry of the funnel plots, and the results of the meta-analysis remained significant after including these two studies (adjusted HR: 1.45, 95% CI: 1.23 to 1.72, *p* < 0.001, *I*^2^ = 89%). The funnel plots for the meta-analysis of hearing loss and AD are shown in Figure 6B. These plots were symmetrical as judged by visual inspection, reflecting low possibility of publication bias. Egger's regression test was not performed for the outcome of AD because only five datasets were analyzed for this outcome.

## Discussion

Results of this meta-analysis showed that hearing loss may be an independent risk factor for dementia in a community-derived general population. Subgroup analyses showed that this association seemed to not be affected by study characteristics such as the diagnostic methods for hearing loss, validation strategy for dementia, follow-up duration, and adjustment of APOE genotype. Although potential publication bias was suggested, a complementary trim-and-fill analysis conducted by incorporating the two hypothesized studies with negative findings showed consistent results with the main meta-analysis. Moreover, we also found a meaningful association between hearing loss and AD. Together, these results suggested that hearing loss may be an independent risk factor for dementia in the general adult population. Whether effective treatment for hearing loss is effective in reducing the incidence of dementia should be further validated.

A previous meta-analysis included 40 studies and suggested that age-related hearing loss may be associated with all-cause dementia (Loughrey et al., 2018). However, the meta-analysis mainly included cross-sectional studies (Loughrey et al., 2018). Accordingly, results of the same meta-analysis failed to indicate a potential temporal relationship between hearing loss and the subsequent incidence of dementia (Loughrey et al., 2018). Besides, univariate studies were included in both of the meta-analyses, which may confound the results. Moreover, the literature search was performed before 2018 in these two meta-analyses, and a number of eligible studies have been published since then (Deal et al., 2017; Golub et al., 2017; Amieva et al., 2018; Ford et al., 2018; Brenowitz et al., 2019; Osler et al., 2019; Vassilaki et al., 2019; Brewster et al., 2021), which have not been evaluated in a meta-analysis.

The studies included in the current work have multiple strengths compared with the previous meta-analyses. First, we only considered prospective cohort studies in our meta-analysis, aiming to minimize the influence of recall biases that could be caused by studies with a retrospective design (Coughlin, 1990). In addition, we only extracted data with multivariate adjusting, which enabled us to indicate a potential independent association between hearing loss and increased risk for subsequent dementia. Since both hearing loss and dementia are age-related conditions (Chern and Golub, 2019), the association between hearing loss and incident dementia may be confounded by age. Our study showed that the pooled HR for the association between hearing loss and incident dementia remained significant after adjusting for multiple variables such as age. However, a recent prospective cohort study using cognitive decline as the main outcome showed that the association between hearing loss and accelerated cognitive decline was non-significant after an additional adjustment for non-linear age effects (Croll et al., 2021). Therefore, the potential role of age underlying the association between hearing loss and incident dementia deserves more investigation. Moreover, up-to-date studies were included in this meta-analysis, and the relatively larger number of datasets enabled us to perform comprehensive subgroup analyses to validate the findings. Results of our meta-analysis applauded the epidemiological link between hearing loss and a higher incidence of dementia. Although the pathological mechanisms remain unknown, some results of previous studies may be helpful in understanding such an association (Griffiths et al., 2020). One popular hypothesis is “the information-degradation hypothesis,” which suggests that hearing loss could lead to impaired auditory inputs, which increases the reliance on cognitive resources and accelerates cognitive decline in elderly population (Pichora-Fuller, 2003). The damage seemed to be long lasting, since pathophysiological studies have suggested that potential deafferentation and atrophy in the auditory system may lead to long-term persistent neuronal reorganization, which can ultimately cause chronic auditory deprivation (Wayne and Johnsrude, 2015). Besides, dementia is a collective term used to describe multiple symptoms, and the association between hearing loss and individual domains of cognitive function may be different (Loughrey et al., 2018). The exact mechanisms accounting for the interaction between hearing loss and elevated risk of dementia deserve further study.

To the best of our knowledge, only two previous meta-analyses have evaluated the association between hearing loss and AD (Zheng et al., 2017; Loughrey et al., 2018). An early meta-analysis combining the results of four prospective cohort studies showed that hearing loss may be related to an increased risk of AD (Zheng et al., 2017). However, one of the included studies evaluated the incidence of mild cognitive impairment (MCI) rather than AD, which severely confounded the finding of the meta-analysis (Zheng et al., 2017). Although the other meta-analysis (Loughrey et al., 2018) did not show a significant association between hearing loss and incident AD in cohort studies, only two cohorts were included in this meta-analysis, which may have been underpowered to indicate a significant association (odds ratio: 1.69 with wide CI: 0.72 to 4.00). Our study included five prospective cohort studies and showed that hearing loss may be independently associated with a higher risk of AD. Future studies are needed to examine the potential role of hearing loss in the pathogenesis and progression of AD.

Results of our study highlight the possibility that alleviation of hearing loss may become a preventative strategy for dementia. The most frequent cause of acquired hearing loss is cochlear damage. Accordingly, amplification with hearing aids or cochlear implants is often effective for these patients (Boisvert et al., 2020; Nijmeijer et al., 2020). A previous study also showed that use of hearing aids attenuated the association between hearing loss and cognitive decline (Amieva et al., 2015). In addition, another prospective study including 15 hearing-impaired elderly patients who were treated with cochlear implantation showed that these patients had significantly improved cognitive function throughout a follow-up period of 1 year (Castiglione et al., 2016). Similarly, a study included 70 hearing-impaired elderly patients (45% with MCI before surgery) and showed that cognitive function of 10% of the participants with MCI became normal in cognitive function 7 years after cochlear implantation (Mosnier et al., 2018). These findings may highlight the possibility that effective treatment for hearing loss could improve cognitive performance and reduce the incidence of dementia (Darwich et al., 2020). Future clinical studies are warranted to validate these hypotheses.

This study also has limitations. First, the meta-analysis was based on data from the study level but not from individual patients, which prevented further analyses on the influence of patient characteristics on the outcome. In addition, diagnosis of hearing loss varied among the included studies, and the association between hearing loss and dementia, according to the severity of hearing loss, could not be determined based on the current meta-analysis. Methods for validation of dementia outcomes also varied among the included studies, which may be a source of heterogeneity. Additionally, we included prospective cohort studies only to reduce any potential bias. However, information from cross-sectional or retrospective studies may be missing. Finally, possible risk of publication bias was raised in the meta-analysis regarding the association between hearing loss and dementia. However, further trim-and-fill analysis suggested that the potential publication bias was not likely to affect the finding.

In conclusion, this updated meta-analysis of prospective cohort studies suggests that hearing loss is likely to be an independent risk factor of dementia, as well as for AD in the general adult population. Clinical studies are needed to clarify whether effective treatment for hearing loss, such as hearing aids and cochlear implantation, can prevent the incidence of dementia in these patients.

## Data Availability Statement

The original contributions presented in the study are included in the article, further inquiries can be directed to the corresponding author/s.

## Author Contributions

ZL, AL, XQ, and XG designed the study. ZL and AL performed literature search, study quality evaluation, and data extraction. ZL, AL, and YX performed the statistical analyses. ZL, AL, XQ, and XG interpreted the results. ZL and AL drafted the manuscript. All authors revised the manuscript and approved its submission.

## Conflict of Interest

The authors declare that the research was conducted in the absence of any commercial or financial relationships that could be construed as a potential conflict of interest.
